# Powered flight in hatchling pterosaurs: evidence from wing form and bone strength

**DOI:** 10.1038/s41598-021-92499-z

**Published:** 2021-07-22

**Authors:** Darren Naish, Mark P. Witton, Elizabeth Martin-Silverstone

**Affiliations:** 1 School of Biological Sciences, Faculty of Environment & Life Sciences, University of Southampton, University Road, Southampton, SO17 1BJ UK; 2 School of the Environment, Geography and Geosciences, University of Portsmouth, Burnaby Building, Burnaby Road, Portsmouth, PO1 3QL UK; 3 School of Earth Sciences, Life Sciences Building, University of Bristol, 24 Tyndall Ave, Bristol, BS8 1TH UK

**Keywords:** Evolutionary ecology, Palaeontology

## Abstract

Competing views exist on the behaviour and lifestyle of pterosaurs during the earliest phases of life. A ‘flap-early’ model proposes that hatchlings were capable of independent life and flapping flight, a ‘fly-late’ model posits that juveniles were not flight capable until 50% of adult size, and a ‘glide-early’ model requires that young juveniles were flight-capable but only able to glide. We test these models by quantifying the flight abilities of very young juvenile pterosaurs via analysis of wing bone strength, wing loading, wingspan and wing aspect ratios, primarily using data from embryonic and hatchling specimens of *Pterodaustro guinazui* and *Sinopterus dongi*. We argue that a young *Sinopterus* specimen has been mischaracterised as a distinct taxon. The humeri of pterosaur juveniles are similar in bending strength to those of adults and able to withstand launch and flight; wing size and wing aspect ratios of young juveniles are also in keeping with powered flight. We therefore reject the ‘fly-late’ and ‘glide-early’ models. We further show that young juveniles were excellent gliders, albeit not reliant on specialist gliding. The wing forms of very young juveniles differ significantly from larger individuals, meaning that variation in speed, manoeuvrability, take-off angle and so on was present across a species as it matured. Juveniles appear to have been adapted for flight in cluttered environments, in contrast to larger, older individuals. We propose on the basis of these conclusions that pterosaur species occupied distinct niches across ontogeny.

## Introduction

The earliest stages of pterosaur life history have long been shrouded in mystery, a fact due mostly to the rarity or absence of eggs and embryos for the majority of taxa, but also to the difficulty inherent in distinguishing hatchlings from small adults,. Comprehensive assessments published since the 1990s have shown that very young pterosaurs can be identified on the basis of both skeletal proportions and the identification of features indicative of osteological immaturity (e.g.^1–5^). More recently, the discovery of eggs, embryos and bone beds have improved our knowledge of the earliest phases of pterosaur growth^6–11^. Among the more surprising conclusions of these studies are that even embryonic pterosaurs were well-ossified and adult-like in skeletal proportions, differing only in a few aspects of bone fusion and proportions. Furthermore, soft tissues preserved in embryos show that flight membranes were present even before hatching^7^.

These lines of evidence indicate that juvenile pterosaurs were capable of powered flight early in life, plausibly within days or hours of hatching. Overall, their development recalls that of precocial sauropsids rather than the altricial offspring of neoavian birds, and it seems reasonable to interpret juvenile pterosaurs as neither nest-bound, nor helpless and dependent upon their parents. Some authors promoting this view have referred to baby pterosaurs as ‘flaplings’ (e.g.^12^).

Some studies, however, cast doubt on the appropriateness of this term. Prondvai et al.^13^ challenged the idea of an early onset of flight in pterosaurs via an analysis of growth rate in the Late Jurassic *Rhamphorhynchus* and argued that pterosaurs grew relatively rapidly at first but slowed in later life. This is the growth style seen in birds, so Prondvai et al.^13^ proposed a bird-like pattern for pterosaurs: they imagined hatchling pterosaurs as flightless and confined to ground-based and climbing behaviours, with flight only becoming possible at about 50% of adult size^13^. It has also been proposed that the Cretaceous anhanguerian *Hamipterus tianshanensis* was potentially flightless at hatching, due to the lack of ossification found in some embryonic wing elements^11^. However, the embryonic bones of other pterosaur taxa are well ossified, so it may be that pterosaurs had differing degrees of precociality at hatching.

It could be argued that a late development of flight in pterosaurs is consistent with the fact that the majority of extant volant vertebrates are incapable of flight in early life. Among the exceptions are certain galliform birds, which have precocial offspring capable of flight on their day of hatching^14^ or within a few weeks^15^. Megapodes (medium-large gallinaceous birds of tropical Asia, Australasia and the western Pacific) have ‘superprecocial’ chicks capable of flying long distances within a few days of hatching^16^. Flight-ready chicks are unusual among birds and are specialised relative to related groups^17^: they are, in fact, so specialised that they may not be ideal models for other tetrapods. The concept of a non-volant phase in pterosaur life history as per Prondvai et al.^13^ is not, therefore, an unreasonable prediction. However, alternative interpretations of the slowing of pterosaur growth exist. It might, for example, be due to the onset of reproductive maturity^18^.

Bennett^4^ described the smallest *Pteranodon* found to date and showed that *Pteranodon* exhibited slow growth. He proposed—based both on measurements collected across *Pteranodon* specimens and on histological sections—that juvenile pterosaurs were precocial, capable of flight from a young age, and grew slowly until they reached adult size. He also suggested that *Pteranodon* occupied more than one niche across ontogeny and that the occupation of a distinct niche by juveniles explained their rarity in the Smoky Hill Chalk Member^4^. He further argued that this was the case for most other pterosaurs too. Hone et al.^19^ showed how skeletal proportions present across the ontogeny of *Rhamphorhynchus* are indicative of precociality and adult-like flight behaviour in hatchlings, and also suggested that different niches were occupied by this taxon across its ontogeny.

Two models for the earliest phases of pterosaur life are thus currently in use. We term these ‘flap-early’ (where the onset of flight occurs at a very young age, maybe even while the animal is still a hatchling) and ‘fly-late’ (where the onset of flight is delayed until later in life). Establishing which of these contrasting interpretations is more likely is relevant to several aspects of pterosaur research, such as understanding pterosaur life history and behaviour, but the issue is of broader relevance with respect to pterosaur ecology and diversity as a whole. If pterosaurs were flight-ready at a very early stage in life, hatchlings could occupy niches that might otherwise be filled by small-bodied pterosaur species or other animals. If so, a single pterosaur species could occupy multiple ecological niches through life, thus potentially lowering pterosaur species diversity, a possibility previously promoted by Bennett^4^.

Currently, arguments over the flight abilities of hatchling pterosaurs have been based on qualitative assessment. Here, we use two quantitative tests to help determine the flight potential of hatchling pterosaurs. Firstly, we modelled the gliding ability of hatchlings to assess whether their wing skeletons were sufficiently developed to support flight. We predict that, if fly-late models are credible, the wings of hatchlings should have been too small to permit efficient gliding. Secondly, we modelled hatchling humeral strength and compared it to the strength of equivalent bones in larger, incontrovertibly volant pterosaurs. The biomechanical demands of powered flight are such that volant animals need strong wing bones even at small size^20^ and, if fly-late concepts have merit, we predict that hatchling humeri would have been weaker than those of flying pterosaurs. These approaches allow us to test a third, ‘compromise’ hypothesis where, if gliding seems possible but hatchling wing bones are relatively weak, we might assume hatchling pterosaurs could glide. In line with our other terminology, we refer to this concept as ‘glide-early’.

*Institutional abbreviations*: AMNH, American Museum of Natural History, New York, USA; BPV, Beijing Museum of Natural History, Beijing, China; BSPG, Bayerische Staatssammlung für Palälontologie und Geologie, Munich, Germany; FGGUB, Faculty of Geology and Geophysics of the University of Bucharest, Bucharest, Romania; IVPP, Institute for Vertebrate Paleontology and Paleoanthropology, Beijing, China; MHIN-UNSL-GEO-V, Museo de Historia Natural, Universidad Nacional de San Luis, San Luis, Argentina; MMP, Museo Municipal de Ciencias Naturales “Galileo Scaglia,” Mar del Plata, Argentina; MOR, Museum of the Rockies, Bozeman, USA; NHMUK, Natural History Museum, London, United Kingdom; NSM, National Science Museum, Tokyo, Japan; RAM, Raymond M Alf Museum of Paleontology, Claremont, California; RBCM, Royal British Columbia Museum, Victoria, Canada; SMNK, Staatliches Museum für Naturkunde, Karlsruhe, Germany; SMNK, Staatliches Museum für Naturkunde, Stuttgart, Germany; SMU, Southern Methodist University, Dallas, USA; TMM, Texas Memorial Museum, Austin, USA; TMP, Royal Tyrrell Museum of Palaeontology, Drumheller, Canada; USNM, National Museum of Natural History, Washington DC, USA.

## Methods

### Taxon sampling

Our investigations centre on pterosaur specimens at or close to ‘hatchling’ status: near-term embryos or recently hatched individuals. Relatively few taxa are represented by such specimens and, of those, only a fraction provide reliable wingspan estimates and humeral cortex data necessary for biomechanical modelling. This latter issue reflects, in part, a failure to record cortical thicknesses in descriptions. These values are (typically) readily available when broken bones are examined and are of great utility with respect to functional and taxonomic studies, so we urge authors to include them in descriptions as a matter of routine.

**Figure 1:**
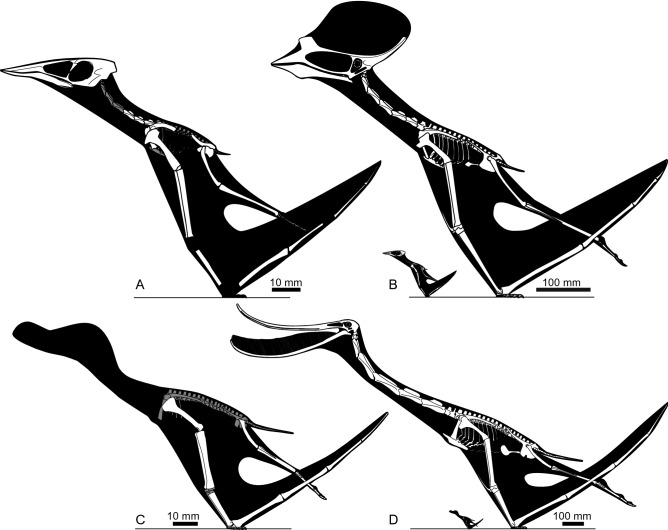
Skeletal restorations of pterosaur taxa (hatchlings and adults) used in this study. (**A**) *Sinopterus dongi* hatchling (based on IVPP V-14377); (**B**) *S. dongi* hatchling compared to adult (adult based on *Sinopterus benxiensis* holotype, BXGM V0011); (**C**) *Pterodaustro guinazui* hatchling (based on MHIN-UNSL-GEO-V 241); (**D**) hatchling compared with adult (adult modified from a skeletal restoration in^74^). White shading indicates well-represented bones requiring no or only minimal reconstruction, grey shading indicates elements which are represented in fossils but are difficult to reconstruct accurately.

Our models of hatchling pterosaurs are based on four specimens of two taxa. Humeral proportions and cortical data from two hatchlings and one embryo of the Cretaceous pterodactyloid *Pterodaustro guinazui* (MHIN-UNSL-GEO-V 237, MHIN-UNSL-GEO-V 241, and MMP 1168; data from^18^ and^21^) permitted us to model two hatchling-age individuals from this species, one with a 0.24 m wingspan and the other with a 0.29 m wingspan (Fig. 1). We also utilised IVPP V-14377, the holotype of *Nemicolopterus crypticus*, to model a hatchling *Sinopterus dongi* (Fig. 1). This Cretaceous pterodactyloid was argued to be a plesion to Ornithocheiroidea (sensu Kellner^22^) by Wang et al.^23^ but has been interpreted elsewhere as a tapejarid azhdarchoid^24^ potentially synonymous with the contemporary Jiufotang Formation tapejarid *Sinopterus*^25^. Brief arguments for IVPP V-14377 being an early-stage juvenile, and part of a Jiufotang tapejarid growth series, were presented by Witton^25^, and we elaborate on these to justify our consideration of this specimen here.

There is no doubt that IVPP V-14377 represents a young juvenile^23^, as is demonstrated by such features as its small size, proportionally enormous orbit, rounded and unfused pelvic bones, poorly defined limb articulations with unfused epiphyses, unfused skull bones, unfused scapulocoracoid, and lack of fusion between the tibia and tarsus (e.g.^2, 3^). Wang and colleagues argued that IVPP V-14377 cannot be a hatchling because it has comparably well-formed and ossified bones, but we note in contrast that embryonic pterosaur specimens, such as those of *Pterodaustro*^6^, have equally well-developed skeletons. Wang et al.^23^ compared the ossification of IVPP V-14377 to juvenile pterosaurs from the Jurassic Solnhofen Limestone, a deposit where diagenetic crystal growth often obscures details of joints^2^ and preservation is often imperfect in smaller specimens. We are thus unsure whether the apparent degree of ossification in IVPP V-14377 can be exclusively linked to ontogenetic factors over those of preservation and posit that IVPP V-14377 could represent a hatchling rather than a young juvenile. Ultimately, it is perhaps unimportant whether IVPP V-14377 is a ‘hatchling’ or ‘very young juvenile’^26^; more critical is that the ontogenetic status of the specimen is not in dispute.

Given the ontogenetic stage of IVPP V-14377, its non-azhdarchoid placement in Wang et al.’s^23^ phylogenetic analysis must be treated with caution. It is well known that the inclusion of juvenile and adult specimens in phylogenetic studies can lead to erroneous phylogenetic placements (e.g.^27^) and result in juveniles being well separated from conspecific adults. Specifically, the fact that juvenile morphologies are often relatively ‘plesiomorphic’ relative to the condition in adults requires that juveniles will not group alongside mature conspecifics but will instead occupy more basal positions^27^. Phylogenetic analyses of Pterosauria are based largely on ‘subadult’ or ‘adult’ specimens and, if IVPP V-14377 does represent a hatchling, its placement in cladograms where most character coding is based on mature specimens may not be reliable.

Comparative anatomy makes it likely that IVPP V-14377 is a misidentified juvenile tapejarid. Despite its size and juvenile status, IVPP V-14377 possesses features that indicate an azhdarchoid, and likely a tapejarid, identity. These include a downturned rostrum, edentulousness, an expanded frontoparietal region, and wing phalanges that decrease in length distally (as preserved, the first and second wing phalanges are of equal length; however, the first phalanx must have been longer in life because it is broken across the shaft, and evidently some distance from the expanded proximal end)^22,28–31^. It is also tapejarid-like in the slender, subvertical lacrimal process of the jugal, inverted ‘piriform’ orbit, the reclined (but not horizontal) occipital region, a jaw joint ventral to the anterior half of the orbit, unwarped deltopectoral crest, long hindlimb, compact metatarsals, short cervical vertebrae with low neural spines, and elongate scapulae^28,30–32^. This combination of features is unknown outside Azhdarchoidea and, within this clade, specific to Tapejaridae. The recovery of IVPP V-14377 among tapejarids in some phylogenetic studies^24^ is in agreement with these observations (although the caveats noted above concerning the inclusion of young juveniles in phylogenetic data sets where character coding is based on adults still apply here).

**Figure 2:**
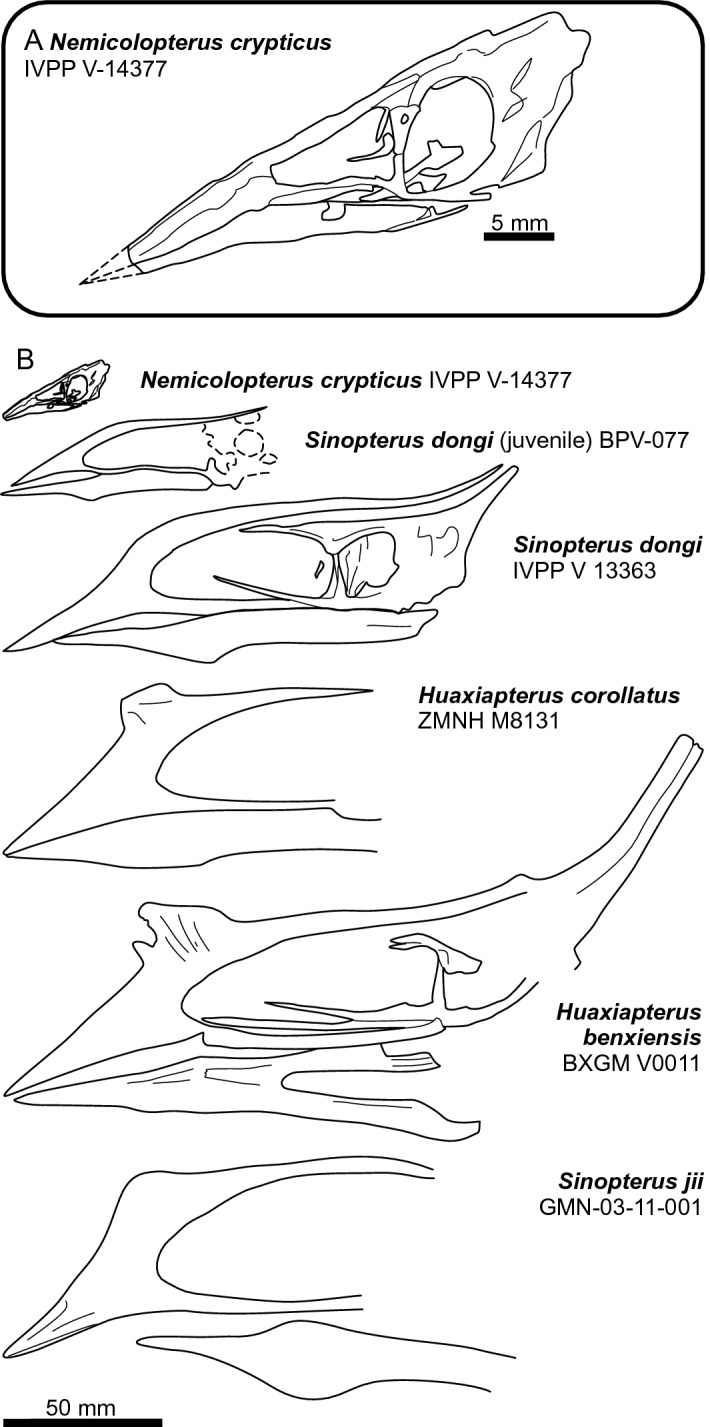
Crania of Jiufotang tapejarid taxa arranged by size. Note progressive change in skull shape and exaggeration of cranial crest in larger specimens, a feature consistent with cranial growth in other pterosaur species. Skulls redrawn from literature.

IVPP V-14377 lacks the rostral and mandibular crests present in many tapejarids (Fig. 2). This is not a problem for a tapejarid identification, however, as pterosaurs, including tapejarids, show correlations between ontogenetic status, size and crest development^8, 9,33–35^. Crests occur more often, and are more developed, in the oldest and largest individuals. The absence of a crest in a tiny juvenile such as IVPP V-14377 is thus unsurprising. Indeed, IVPP V-14377 conforms well to the correlation between crest development and skull size in tapejarids from the Jiufotang Formation (Fig. 2). The smallest confirmed tapejarid from this formation—BPV-077—lacks a rostral crest and has a poorly developed dentary crest^36^, while progressively larger skulls show deeper crests and longer crest bases. This indicates that even smaller, younger tapejarids would have crests no larger than that of BPV-077 and might even lack crests altogether: the condition present in IVPP V-14377. BPV-077 is about twice the size of IVPP V-14377 but half the size of the next largest named specimen (IVPP V13363) and offers a reasonable ‘intermediate’ between the condition of IVPP V-14377 and that of mid-sized Jiufotang tapejarids.

**Figure 3:**
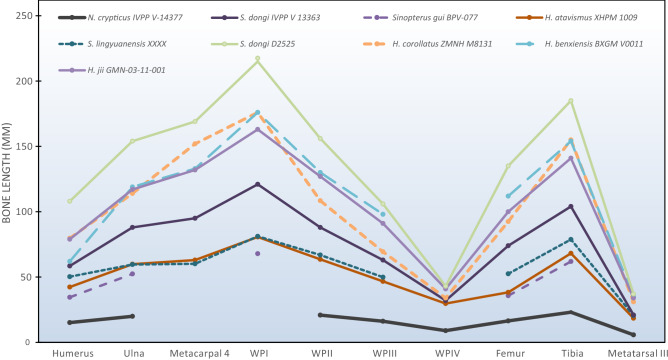
Limb ratios of Jiufotang tapejarid specimens. Limb metrics taken from descriptive papers of listed taxa.

IVPP V-14377 also matches trends of Jiufotang tapejarid limb scaling, as shown in Modified Nopcsa Curves (sensu Bennett^37^) of limb proportions (Fig. 3). IVPP V13363 shows the same curve structure as the Jiufotang tapejarids but not the exaggerated curves characterising the biggest specimens. Smaller and mid-sized specimens—such as BPV-077 and IVPP V13363—show intermediate curve shapes linking these extremes. This same trend of curvature is seen in other pterosaur growth series, like those of *Rhamphorhynchus* and *Pterodactylus*^2,38^. We also found that humerus-derived scaling regimes of eight Jiufotang tapejarids predicted limb metrics highly similar to those of IVPP V-14377, within 4.2 mm in all but one instance (length of wing phalanx IV) (Fig. 3). The error bars on these estimates are high given the large size range of the specimens concerned but, nevertheless, these data suggest that a hatchling Jiufotang tapejarid would be very similar in proportions to IVPP V-14377.

To summarise, IVPP V-14377: (1) is clearly a young juvenile; (2) has anatomical characteristics unknown outside of Tapejaridae, and is consistent with this group in all significant attributes; (3) meets predictions of tapejarid scaling trends in size and anatomy, such as cranial crest and limb proportions; and (4) occurs in a formation which yields abundant tapejarid remains. These criteria indicate that IVPP V-14377 is a juvenile tapejarid, and we consider this option more parsimonious than it representing a diminutive, non-azhdarchoid taxon. This alternative requires IVPP V-14377 to be highly convergent with tapejarid morphology, coincidentally matching projected tapejarid ontogenetic anatomies, and coincidentally to be the right size and ontogenetic status to be a juvenile of a Jiufotang tapejarid species. This is not impossible but less likely than our preferred interpretation.

Comparing IVPP V-14377 to specific Jiufotang tapejarid taxa is complicated by the near certainty of their being taxonomically oversplit. This formation has two tapejarid genera and six species which are generally considered valid (or at least not widely contested): *Sinopterus dongi*, *S. jii*, *S. lingyuanensis*, *Huaxiapterus corollatus*, *H*. *benxiensis* and *H. atavismus.* Virtually all proposed diagnostic features of these taxa pertain to limb bone ratios, details of crest anatomy and skull proportions, features known to correlate with size changes across pterosaur ontogeny^1,2, 8,9,35,38,39^. We encourage a detailed taxonomic revision which tests the status of these putative taxa based on first-hand examination but conclude—based on our presented trends of limb and crest scaling in these specimens—that most or even all of them (including IVPP V-14377) are synonymous and represent growth stages of *S. dongi*^40^. They certainly vary no more than specimens recovered from pterosaur bone beds (e.g.^8,9^) or taxa represented by growth series^2,21,35,38,41^. ZMNH M8131 (the holotype of *H. corollatus*) is an apparent outlier, however, in having especially long wing metacarpals and a high femur/tibia ratio and may represent a second taxon. Our proposals require further testing; these should incorporate the production of improved descriptions and illustrations of the Jiufotang tapejarid holotypes (all the taxa discussed here remain tersely described and figured), and searches for histological indicators of maturity.

### Glide analysis

Studies on living gliders (e.g.^42^) show strong correlations between wing loading and glide performance, highlighting that strong gliding performance reflects optimisation of relationships between wing area, body mass, and gravity^43^. Glide performance is thus relatively easy to predict for extinct fliers if these parameters can be predicted.

We modelled hatchling pterosaur masses using a relationship predicted between pterosaur wingspan and body mass (^44^; R^2^ = 0.983). Comparable datasets are available (e.g.^45^) but the predicted relationships between mass and wingspan are similar enough that both datasets give similar results^46^. The scaling equation used here is:

1 $$M =0.557b^{2.5520}$$

where *M* is body mass (kg) and *b* is wingspan (m) (though see^47^ and^48^ for discussions on the use of this mass estimation method). Wing area is difficult to calculate for pterosaurs because wing membrane fossils are rare, difficult to interpret precisely^49^, and generalisations made about some pterosaurs may not apply to all taxa. We thus estimated wing area using a regression between wingspan and wing area from 90 extant bird species (R^2^ = 0.969; we aim in future to expand this dataset via the addition of bats). This method may obscure some nuances of pterosaur wing anatomy, but nevertheless allows for repeatable and objective calculations of this metric as well as predictions of scaling trends that might be overlooked through subjective predictions of wing size. We used the following equation to predict wing area:

2 $$S = 0.1184b^{1.7802}$$

where *S* is wing area (m^2^). From these equations, other parameters necessary to estimate glide performance—aspect ratio, wing loading, body weight—are readily computed using conventional calculations.

Glide performance was modelled using *Flight* v. 1.24^50^. This program is designed for the modelling bird flight but (with minor modification) can predict the flight performance of membranous fliers such as pterosaurs (Habib pers. comm. 2010; see^51^ for details). Necessary adjustments include limiting span reduction in flight to 80% of wingspan (Bstop values of 6.0) to reflect the risk of membranous wings fluttering at reduced span (on this point, we are cognisant of structures which may have reduced or prevented flutter in pterosaur membranes^52^). Maximum lift coefficient was set to 2.2, the largest measured from living birds, reflecting the likelihood that membranous pterosaur wings exceeded feathered wings in this respect^51^.

### Bone strength analysis

We focused our bone strength assessments on pterosaur humeri as this bone—the proximal bone of the primary limb powering takeoff—should exhibit the strongest mechanical signal of flight capacity^20^. As per Witton and Habib^51^, we performed bending strength assessments using standard beam loading calculations. Pterosaur humeri have complex proximal and distal ends but are hollow tubes in their diaphyseal region and thus amenable to beam loading analysis^20,51^. We modelled three hatchling pterosaur humeri and 22 humeri of pterosaurs representing larger, older individuals. Our non-hatchling dataset comprises pterosaurs representing the effective size range of the entire clade (0.23–10.4 m wingspans) and samples multiple parts of the pterosaur tree (Table 1).

**Table 1:** Parameters and results of humeral relative failure force (RFF) analysis.

Identification	Specimen number	Wingspan (mm)	Load (N)	Length (mm)	Diaphysis diameter (mm)	Cortical thickness (mm)	R/t	Section bone area (mm^2^)	Area moment of inertia	Section modulus	Maximum stress (Mpa)	Relative failure force (RFF)
*Pterodaustro guinazui* (hatchling)	MHIN-UNSL-GEO-V 237	244	0.16	16.5	1.8	0.10	8.90	0.54	0.20	0.23	11.31	14.32
*Pterodaustro guinazui* (hatchling)	MHIN-UNSL-GEO-V 241	290	0.24	19.2	2.1	0.10	10.35	0.80	0.38	0.37	12.55	12.91
*Sinopterus dongi* (hatchling)	IVPP V-14377	250	0.17	15.2	1.8	0.10	9.10	0.54	0.20	0.22	11.19	14.48
*Anhanguera piscator*	NSM-PV 19892	4400	224.55	255.0	32.1	1.11	14.46	108.07	12,989.86	809.34	70.75	2.29
*Anhanguera* sp.	SMNK 1133	5628	417.06	290.0	40.5	0.66	30.66	82.54	16,356.85	808.34	149.62	1.08
*Anurognathus ammoni*	Private specimen (Bennett 2007)	226	0.13	18.4	1.5	0.20	3.65	0.79	0.16	0.22	10.65	15.21
Azhdarchidae indet	RAM 18659	3084	91.84	173.3	21.3	1.03	10.32	65.43	3352.48	315.53	50.44	3.21
Azhdarchidae indet	TMP 1992.83.4	4596	250.57	265.0	40.2	0.86	23.39	106.37	20,618.72	1025.04	64.78	2.50
Azhdarchoidea indet	NHMUK PV 2353	1733	21.53	105.9	12.2	1.05	5.82	36.88	581.28	95.06	23.99	6.75
Azhdarchoidea indet	RBCM.EH.2009.019.0001	1405	12.70	75.0	7.2	1.04	3.48	20.22	99.60	27.55	34.58	4.68
Azhdarchoidea indet	Wessex Azhdarchoid	2829	73.91	158.4	17.7	1.40	6.30	71.47	2376.62	269.31	43.47	3.73
*Bennettazhia oregonensis*	USNM 11925	3221	102.47	180.0	19.8	1.46	6.80	83.90	3555.55	358.88	51.39	3.15
*Brasileodactylus* sp.	AMNH 24444	2934	80.98	166.2	21.2	0.67	15.81	43.17	2272.45	214.58	62.71	2.58
*Germanodactylus rhamphastinus*	BSPG 1977 XIX1	856	3.65	39.7	4.1	0.70	2.90	7.39	10.88	5.36	27.04	5.99
Glen Rose humerus	SMU 72547	2338	45.75	197.0	24.7	1.70	7.26	122.84	8166.92	661.29	13.63	11.89
*Hatzegopteryx thambema*	FGGUB R1083	10,400	1955.39	544.0	101.7	5.50	9.24	1661.87	1,927,945.47	37,921.82	28.05	5.78
*Montanazhdarcho minor*	MOR 691	3235	103.58	182.3	22.5	0.70	16.07	47.94	2850.85	253.41	74.52	2.17
Ornithocheiridae indet	NHMUK PV R 39	3917	167.61	212.8	26.8	0.95	14.08	77.03	6422.96	480.04	74.29	2.18
Ornithocheiridae indet	NHMUK PV R 1357	5331	363.90	276.9	36.0	1.17	15.37	127.91	19,385.37	1077.86	93.48	1.73
"*Rhamphocephalus*" sp.	NHMUK PV R 40126	948	4.72	58.4	6.3	0.86	3.65	14.62	54.83	17.49	15.78	10.26
*Rhamphorhynchus muensteri*	RAM 14522	910	4.26	32.0	4.0	0.48	4.17	5.31	8.37	4.19	32.55	4.98
*Rhamphorhynchus muensteri*	SMNS 9620	1000	5.40	35.9	3.6	0.51	3.49	4.89	5.84	3.28	59.01	2.75
*"Santanadactylus pricei"*	BSP 1980 I 122	3150	96.86	174.0	16.0	1.00	8.00	47.12	1331.25	166.41	101.28	1.60
*Quetzalcoatlus* sp.	TMM 41916	4660	218.93	250.0	(data from Witton and Habib^51^)							4.78
*Quetzalcoatlus northropi*	TMM 41450–3	10,400	2451.66	544.0	(data from Witton and Habib^51^)							2.31

Our models assume a circular cross section of the mid-length humeral diaphysis. This is a simplification of the true oval shape of this region but is necessary given the fact that many pterosaur humeri are crushed with shaft diameters only measurable in one aspect. Where undistorted 3D specimens were available, we averaged width and height measurements to reflect their condition more accurately. Humeral dimensions and cortex thicknesses of each humerus were taken from specimens, thin sections, and published photographs and measurements. For *Pterodaustro* we had to take cortical thicknesses from a sectioned 16 mm long humerus (MMP 1168) and shaft dimensions from other specimens (MHIN-UNSL-GEO-V 237, MHIN-UNSL-GEO-V 241), as no hatchling *Pterodaustro* humeri provide both sets of necessary data.

We calculated second moment of area (*I*) for each section using:

3 $$I = \pi \left( {d_1^4 - d_2^4} \right)/64$$

where *d*_1_ is the total bone diameter, and *d*_2_ represents the diameter of the internal bone cavity.

Bone stress was modelled using cantilever-style loading, where one end of the humerus is fixed, the length of the bone equals the moment arm, and stress values reflect those experienced at the supported end of the bone. This simplifies loading regimes experienced in life but provides a useful metric to compare bone strength^51^. Stresses (*σ*, Mpa) experienced at the supported end of the humerus were calculated with:

4 $$\sigma = WL/Z$$

where *W* is the weight loaded onto the bone (N), *L* is bone length (mm), and *Z* is section modulus (second moment of area/distance to neutral axis of vertebra). Pterosaur body weights, specific for each model and calculated from the wingspan/body mass equations of Witton (^44^; also see above), were used to deduce *W*. This allows for determination of Relative Failure Force (RFF; bone failure force, in bending, divided by total body weight) for each humerus and permits comparison of bending strength across a range of differently sized individuals^50^. We followed Palmer and Dyke^53^ in assuming a Young’s Modulus of 22 Gpa, a value consistent with avian long bones and a reasonable proxy for pterosaur bones. Our predicted relationship between Young’s Modulus and yield stress in tension is 162 Mpa, following^53^ and^54^.

## Results

### Glide performance

**Figure 4:**
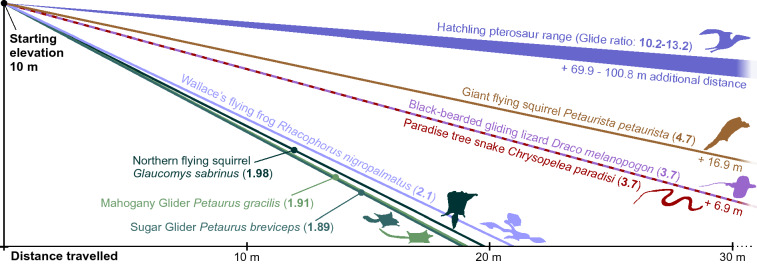
Best glide angles of gliding tetrapods and hatchling pterosaurs. Hatchling pterosaur glide angles represent the range of results obtained for all hatchlings modelled in this study (both minimum sink and best glide values), gliding tetrapod values obtained from^42,55–57^. See Table 2 for predicted wing parameters of hatchling pterosaurs.

Comparison of our predicted hatchling glide performance (Table 2) and wing parameters to modern gliding tetrapods suggests hatchling pterosaurs were superior gliders. Glide ratios (distance travelled vs. altitude loss) of living tetrapod gliders range from c. 2–4.7^42, 55–57^ but those of our hatchling models are 10.2–10.5 at minimum sink speeds and 12.8–13.2 at best glide speeds (Fig. 4). Hatchling pterosaurs thus show capacity for sustained, far-reaching glides that far surpass the glide ranges of even adept living gliders. Gliding performance is largely correlated with decreasing wing loading, and we ascribe much of the hatchling’s glide ability to their large wing skeletons. Even though our predicted hatchling masses are similar to those of extant gliders (such as the large *Draco* species *D. fimbriatus,* 15.8–21.6 g^42^), their wing loading is much lower (measured as 22.7–24.2 N/m^2^ in *fimbriatus*^42^, vs. 15.83–17.88 N/m^2^ in the hatchling dataset). Evidently, hatchling pterosaur wings were sufficiently proportionate to their body size to permit gliding behaviour, casting strong doubt on a ground-bound phase of early life history.

**Table 2:** Hatchling and adult pterosaur wing parameters used in glide analysis.

Taxon	Ontogenetic status	Anatomical parameters						Minimum sink				Best glide			
		Wingspan (m)	Mass (kg)	Body weight (N)	Wing area (m^2^)	Aspect ratio	Wing loading	True air speed (m/s)	Sink rate (m/s)	Glide ratio	Lift coefficient	True air speed (m/s)	Sink rate (m/s)	Glide ratio	Lift coefficient
*Pterodaustro guinazui*	Hatchling	0.244	0.02	0.15	0.009	6.62	16.57	4.40	0.42	10.50	1.47	6.80	0.52	13.20	0.72
*Pterodaustro guinazui*	Hatchling	0.290	0.02	0.23	0.013	6.53	17.88	4.60	0.44	10.40	1.46	7.20	0.55	13.10	0.70
*Sinopterus dongi*	Hatchling	0.250	0.02	0.16	0.01	6.23	15.83	4.40	0.43	10.20	1.42	6.80	0.53	12.80	0.69
*Pterodaustro guinazui*	Adult	3.000	9.19	90.19	0.84	10.80	107.75	10.30	0.70	14.70	1.72	15.60	0.85	18.30	0.85
*Sinopterus dongi*	Adult	1.902	2.87	28.19	0.37	9.72	75.80	8.80	0.64	13.70	1.65	13.40	0.78	17.10	0.82

Our gliding calculations emphasise differences between gliding animals and hatchling pterosaurs, and thus raise the possibility that hatchlings were powered fliers. Gliding tetrapods are characterised by short, broad wings with aspect ratios between 1.2 and 2.3^58^: our calculations predict that pterosaur hatchlings had long, narrow wings with aspect ratios of 6.1–6.5, values consistent with those of powered fliers (avian AR = 5–12). The low aspect ratios of gliding tetrapods are disadvantageous from the perspective of long-distance fliers as they compromise lift-drag ratios^58^, but are ideal for short-range, unpowered fliers. Their advantages include high lift-coefficients at low speeds, wing usage without stall at high angles of attack, and minimal investment in metabolically expensive, anatomically expansive gliding specialisations^58,59^. Such wings are not rudimentary flying surfaces, but optimised and specialised for species that travel moderate distances between elevated positions. We can thus view the higher aspect wings of hatchling pterosaurs as inconsistent with a climber-glider lifestyle, and instead suited for sustained flight in open settings. In this respect, details of hatchling pterosaur wing form are inconsistent with the glide-early hypothesis, but consistent with a flap-early strategy.

### Bone strength analysis

**Figure 5:**
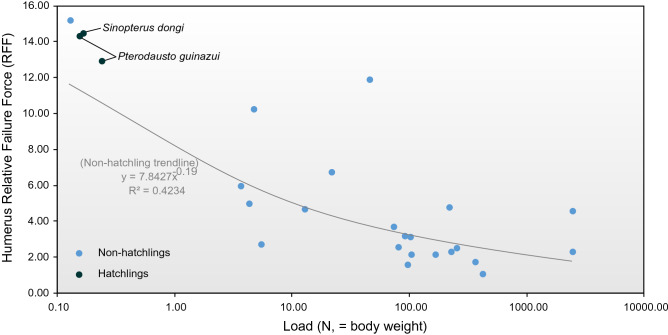
Relative Failure Force (RFF) relative to body weight (N) of adult and hatchling pterosaur humeri. See Table 1 for bone parameters used in calculating humeral RFF.

Our RFF results (Table 1, Fig. 5) show wide variance in pterosaur humeral strength and a predictable correlation with body size^60^. Non-hatchling RFFs range from 1.08 to 15.21, the highest value being from an immature (though post-hatchling) specimen of *Anurognathus ammoni* (wingspan 0.226 m, estimated body weight of 0.13 N) and the lowest from a specimen of *Anhanguera* (estimated wingspan 5.6 m, estimated body weight 417 N). Average RFF for the non-hatchling set is 5.69 (± 3.61 SD) and we take this as an average strength value required to withstand flight demands. The three hatchling humeri models showed RFFs of 12.9–14.48, values over twice our strength threshold and approaching the upper end of the non-hatchling strength range. Hatchling pterosaur humeri are—proportionate to body weight—therefore among the strongest of any pterosaur. A caveat is that we assume the same bone density for both hatchling and adult pterosaurs, and thus the same material properties (e.g. Young’s Modulus). This may be worth further investigation, but the discrepancy between hatchling and adult RFF necessitates that hatchling bone density would need to be considerably lower to reduce wing bone strength to sub-flightworthy levels.

**Figure 6:**
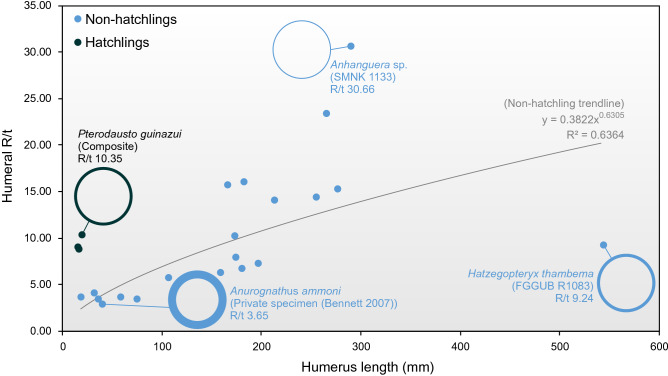
Humeral R/t (bone radius/cortex thickness) ratios in hatchling and adult pterosaurs.

We ascribe the strength of hatchling humeri to two factors. The first is that, other than absolute size, their skeletal metrics are indistinguishable from those of larger pterosaurs. Their linear proportions relative to body size and wing length are the same as those of larger animals (Fig. 1), and their R/t values (proportion of diaphyseal cavity to cortex) are as high (8.9–10.35) as those of larger pterosaurs (Fig. 6)^54,61^. The strength of pterosaur humeri has been well established through biomechanical comparison to other pterosaur bones as well as those of extant analogues^20,51^ and our data suggests that this was present in even the youngest pterosaurs.

The second factor is that the low body masses of hatchlings place minimal loading on their wing skeletons. Scaling relationships mean that hatchling wingspans c. 3% those of the largest pterosaurs in our dataset still result in masses well under 1% of those of the largest animals. Our recovery of high RFFs in a juvenile *Anurognathus*, and a general trend of decreasing humeral RFF against body size (Fig. 5), suggests that this result is not limited to hatchlings but likely applies to any small pterosaur, adult or otherwise.

These indications of extremely strong humeri in hatchlings provide further reason to reject ‘fly-late’ and ‘glide-early’ models, and are consistent with notions that hatchlings were adapted for powered flight: that is, the ‘flap-early’ model. The robustness of pterosaur humeri has been linked to the demands of forelimb-assisted launch^20,62^, and the exceptionally high humeral bending strengths of hatchlings leaves little doubt about launch capacity. Launch is one of the most demanding phases of flight^20^ so it follows that, if hatchling wing bones were sufficient to withstand launch stresses, they would meet any subsequent flight demands (e.g. flapping, manoeuvring). Indeed, the atypically high strength of the hatchling wing skeleton makes it possible that strenuous flight activities were practised on occasion, these potentially including rapid changes in speed and direction, maintenance of slow flight, or hovering. The variation in hatchling wing proportions suggests that the nuances of flight varied between taxa, and further research into hatchling limb morphology may reveal specific predictions of flight capacity. Could humeral strength in hatchling pterosaurs be linked to some non-flight role for the forelimbs (like digging, or forelimb-dominated leaping)? The fact that these animals possessed wing bone proportions much like those of adults strongly suggests, we argue, that flight capability best explains the humeral strength of hatchling pterosaurs.

## Discussion

Data from both the glide and bone strength analyses are consistent with interpretations of hatchling pterosaurs as powered fliers and suggests that—at least in regard to skeletal structure—pterosaurs were flight-ready from the moment they hatched. We also draw attention to the fact that the majority of hatchling pterosaurs have large deltopectoral crests, indicating the potential for large flight muscles to operate their wing skeletons, and the recovery of flight membrane fossils with embryonic specimens^12^ as further evidence of hatchling soft-tissues being flight-ready. Wang et al.^9^ stated that late-stage embryos of the Early Cretaceous pterosaur *Hamipterus* had poorly developed deltopectoral crests, and therefore had poorly developed flight muscles, meaning they were not capable of flight as hatchlings. However, we disagree with this premise. Firstly, the deltopectoral crest of *Hamipterus* looks similar in morphology to those in the embryos of *Pterodaustro*^10^, and not substantially different from those in some subadult ctenochasmatids [e.g.^63^]. Secondly, because flight muscle volume scales with a similar exponent to body mass, a smaller deltopectoral crest does not preclude flight in hatchlings. Hatchlings should have had considerably smaller flight muscle volumes than even juveniles and subadults, which allows for smaller flight muscle correlates.

The flight adaptations of hatchling pterosaurs cast doubt on the idea that pterosaur growth rates changed in response to the onset of powered flight^13^. This hypothesis requires hatchlings to develop and maintain flight-capable wings several years before they were used (assuming the pterosaur growth rates of Chinsamy et al.^18^), a possibility which seems unlikely given the perspective of wasted energy investment, and one which contrasts with the pattern seen in those extant vertebrate fliers incapable of flight at hatching or birth (in such animals, wing development is delayed). Our data supports the concept that other changes in physiology or behaviour, such as the onset of reproductive behaviours^17^, provide more likely explanations for changes in pterosaur growth rates.

It is difficult if not impossible to state precisely when hatchling pterosaurs began to fly given that presently unknowable factors—possible parenting behaviours, hatchling coordination, last-minute soft-tissue development, the nature of nesting environments and so on—likely influenced this, as they do in living megapodes^14^. However, the resource investment that seemingly contributed to the development of hatchling flight anatomy favours earlier, rather than later, development of volancy, and we do not predict a protracted delay between hatching and flying.

Our results are further evidence of pterosaur hatchlings being highly precocial and potentially capable of living independently of their parents (e.g.^4, 12^). However, we note that precociality does not necessarily correlate with an absence of parenting, as many precocial tetrapod species (including mammal, palaeognath, galliform and crocodylian species) receive post-hatching/post-birth parental care, even including direct or assisted parental feeding (e.g.^64^). In these species, precociality means that the juveniles can follow and keep pace with their parents and other conspecifics; they do not live independently of them. Hone et al. made essentially the same point^19^. Thus, an alternative interpretation of flightworthy anatomy in juvenile pterosaurs is that it perhaps gave hatchlings the capability to follow and associate with their parents or other conspecifics. Parental care is less likely—though not completely disallowed—for those species known from deposits where the fossils represent animals of a restricted range in age and size (e.g. the Niobrara Formation, where the majority of specimens are large and mature^1, 4,33^); it remains conceivable for species known from formations yielding specimens of varying age (e.g. the Solnhofen Formation^2,3,13,37,38^) or bone beds containing a range of growth stages^8,9^. Overall, the variation in the pterosaur record suggests that there may be no single ‘universal’ pterosaur parenting strategy: as with living animals, a range of strategies might have existed.

### Pterosaur growth stages: consequence of scaling, or adaptive opportunity?

The volant potential of hatchling and other young juvenile pterosaurs raises questions about changes in flight behaviour across growth, an area previously discussed for *Pteranodon* and other pterosaurs^4, 12^. Hatchling pterosaurs are, in some respects, proportionally similar to their parents, but fundamental aerodynamic relationships between flight and body size mean that flight performance would have differed between younger, smaller individuals and their larger, older counterparts^65^. Among modern animals, these relationships are best demonstrated by flying species which exhibit variation in size: galliforms with flight-ready fledglings show size-correlated flight performance variance that influences ecology and behaviour^15,66^. The comparison between galliforms and pterosaurs is limited because galliforms are primarily terrestrially adapted, heavy-bodied, have high wing-loadings and are adapted for burst flight and rapid takeoff—a marked contrast to the pterosaurian strategy where emphasis is on low or moderate wing-loading and efficient, sustained flight^44,45,51^. Nonetheless, those changes in wing performance present across galliform ontogeny verifies the fact that body scaling influences volant animal ecology and behaviour.

**Figure 7:**
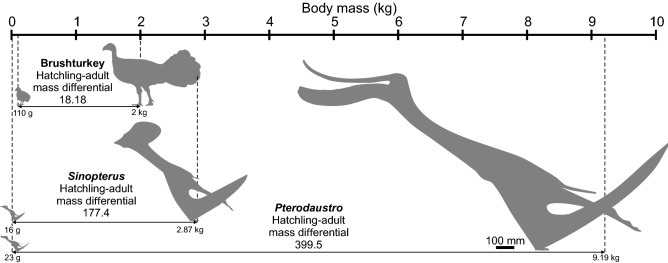
Hatchling-adult body mass differentiations in precocial flying tetrapods. Pterosaur mass differentials are considerably larger than those of any extant precocial fliers, which we predict as having a significant impact on the flight performance and ecology of their growth stages.

To demonstrate the impact such size change could have on pterosaur flight across ontogeny we modelled the wing parameters of adult *Sinopterus* (1.9 m wingspan) and *Pterodaustro* (3 m) and compared them with those predicted for hatchlings using *Flight 1.24* (Table 2). These models are simple in that they do not incorporate such differences as wing structure, body proportions and so on, which have major influences on flight performance^65^. However, they show how basic scaling might have impacted pterosaur flight behaviour across ontogeny, a change that was likely significant due to its magnitude (Fig. 7). Modern size-correlated changes to flight performance occur at relatively small size differentials. For example, the Brush turkey (*Alectura lathami*, Megapodiidae) has relatively large chicks that hatch at 110 g and adults of no more than 2000 g, a magnitude difference of 18.18)^66^. For large pterosaurs, the mass differentials between hatchlings and fully grown individuals was much greater. Our estimated mass of a 0.29 m wingspan *Pterodaustro* hatchling is 23 g, whereas a 3 m span adult is modelled at 9.19 kg: a differential of almost 400. *Sinopterus* is predicted to have a lesser differential of 177.4 on account of its smaller maximum body size (predicted size range of 0.25–1.9 m wingspan; 0.016–2.87 kg body mass), but this is still significantly higher than in birds like megapodes.

We calculate that large individuals of *Sinopterus* and *Pterodaustro* had higher wing loadings than juveniles but also higher aspect ratios; accordingly, larger individuals had higher flight speeds, higher lift coefficients and improved glide ratios (Table 2). These characteristics are ideal for low manoeuvrability flap-gliding and rapid travel across open regions, and match expectations for comparably sized birds^67^ and other large pterosaurs^51^. These predictions accord with general trends seen in other scaling regimes, such as reduced humeral RFFs at larger size (Fig. 5 and Table 2^51^) and the increased need for soaring at larger wingspans, on account of reduced power available for flapping^68^.

With reduced flight speeds and lesser glide performance (Table 2), hatchling *Sinopterus* and *Pterodaustro* would not have been as efficient as their parents at long-distance travel. Refuelling stopovers and similar behaviours would likely be needed should hatchlings have attempted long journeys, whereas these would be less necessary or unnecessary in larger, older pterosaurs. Importantly, the wings of hatchlings were better suited to other forms of flight. Lower aspect wings perform better at elevated angles of attack^59^; their reduced power requirements could sustain flapping over longer periods; their proportionally stronger skeletons could accommodate rapid shifts in centre of mass, and thus allow greater agility^60^; and their reduced wing loading would allow for slower flight speeds. Moreover, launch velocity requirements (and thus energy investment) scale with body mass^68,69^, meaning that smaller pterosaurs had an energetic advantage during takeoff. Collectively, these attributes might have rendered juvenile pterosaurs more dynamic fliers than their parents, better able to switch between aerial and terrestrial locomotion, better suited to executing sudden changes in direction and velocity, and capable of nimbler flight in complex environments.

Bennett^4^ proposed a similar idea of ontogenetic niches for *Pteranodon*: he posited that younger juveniles spent time in terrestrial environments while subadults and adults spent more time at sea. He further suggested that hatchlings and juveniles could have been found near lakes, wetlands or coasts, where foraging excursions would have been brief relative to those of older animals, and that their ability to fly further, and for longer periods, happened in step with their tendency to become increasingly marine as they matured. This view potentially explains why relatively few *Pteranodon* juveniles and hatchlings are found in the sediments of the Western Interior Seaway, a major contrast with the remains of adults^4^.

As argued previously by Bennett^4^, these data are generalised enough to assume they could apply, broadly at least, to pterosaurs of all lineages, and we propose that it may have been common for pterosaurs to vary in flight behaviour as they grew. If so, future studies might do well to consider the significance of the ontogenetic status of any given specimen used in the modelling of flight behaviour: the flight performance of a single individual may not be representative of all behavioural nuances available to that species, particularly if it represents an extreme on the ontogenetic spectrum of the species concerned.

Were these distinctions in flight significant to pterosaur lifestyle and ecology? Precociality in pterosaurs might have allowed them to occupy distinct niches^4,12^ but the importance of flight with respect to ontogenetic niche-shifting has not been explored in detail. The changes in wing form that pterosaurs underwent during growth might not be viewed as strictly adaptive because much of their effect on flight reflect unavoidable laws of scaling. However, this is not to say that pterosaurs could not have exploited these changes in their flight behaviour at different body sizes. For instance, the high power:mass ratios of juveniles and potential for both steeper climb phases and more dynamic flight might have assisted with the avoidance of predators, allowing small pterosaurs to rapidly escape even when in cluttered settings. The same traits may have allowed hatchlings and small juveniles to chase more nimble prey than their parents, as well as fly in complex, heavily vegetated environments off-limits to larger, less manoeuvrable adults. This potential for flight in vegetated environments may, in turn, have afforded a wealth of hiding spots and foraging opportunities for small pterosaurs. Larger, older individuals—less able to practise especially dynamic flight or exploit cluttered habits—may have found the increased travel potential of larger size advantageous for the crossing of open spaces and exploitation of widely spaced food sources.

**Figure 8:**
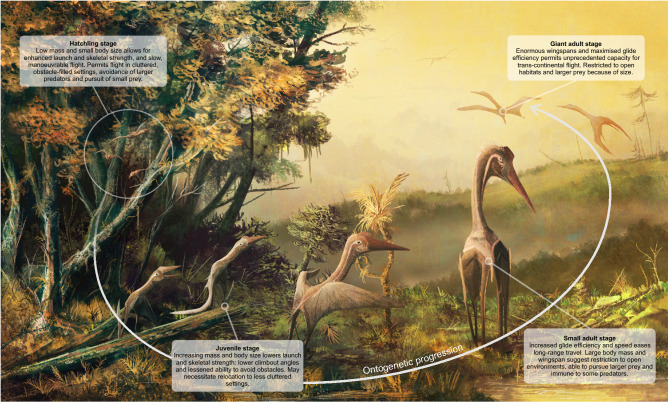
Visual summary of how basic, size-dependent flight parameters (wing loading, wingspan and aspect ratio) could have influenced pterosaur ecology throughout ontogeny. The animals shown here are giant azhdarchids, species which likely had the largest ontogenetic mass differentials of any pterosaurs (see text) and thus potentially the broadest ecological range across their various growth stages. Azhdarchids were primarily terrestrial pterosaurs^75^, which is reflected in this figure, though the environments and points made here are generalised: they do not expressly pertain to any azhdarchid taxon. Ontogenetic niche exploitation may have differed in other environments.

These concepts are potentially more complex than the hatchling vs. adult dynamic presented here. Some pterosaurs were small throughout life^12,24^ and distinctions in flight performance may have been less dramatic between ontogenetic extremes. Conversely, many pterosaurs were large animals with small offspring^70^, creating large intraspecific size ranges and the potential for multiple potential niches. It is not inconceivable that a generalist terrestrial species like *Sinopterus dongi* (see^25,28,71^ for discussion of tapejarid habits) could begin life as an agile, 0.25 m wingspan forest flier with a diet composed of insects and seeds before transitioning to an animal of peripheral woodlands once agility and size precluded flight in densely vegetated settings, finally committing to open environments and associated dietary options when maximum flap-gliding potential was reached at a wingspan of 1.8 m. Larger pterosaurs could have had even more stages and complications in their life histories, especially since egg and hatchling size scales with a low exponent against adult body size (^72^, supplementary data). For the largest pterosaurs, which seem supremely capable of long-distance flight, animals that start life as c. 1 m span, small-bodied foragers could transition to 10 m span continent-hoppers and major predators in some ecosystems (Fig. 8^51,73^).

Fundamental aspects of wing form are in accordance with the idea that pterosaur growth stages could occupy discrete niches, and further research into this idea is encouraged. We stress that the concepts outlined here are hypothetical: locomotion is only one component of animal ecology, and the concept of pterosaurs of differing growth stages occupying a range of habitats and foraging strategies requires support from other studies, particularly those concerning dietary adaptations and the specifics of non-volant locomotion.

## Conclusions

The concept that pterosaurs were flightworthy from the moment of hatching is not new^4,6,7,12,19^ but is supported here by the first quantified assessment of hatchling pterosaur flight performance and wing strength. We are doubtful of the suggestion that changing pterosaur growth rates were linked with the onset of powered flight^13^. Our findings present pterosaurs in contradictory lights. On the one hand, they highlight how conservative these animals were throughout their lifespans: juveniles not only resembled adults, but shared fundamental elements of skeletal structure and function with them. On the other hand, we have also demonstrated how distinct pterosaurs might have been at the beginnings and ends of their lives due to the profound effect of size changes on their ecology and flight behaviour. Our understanding of pterosaur aerodynamics and lifestyle through the perspective of ontogeny is currently limited but promises to be a rich field for future research.
