# Author Correction: Powered flight in hatchling pterosaurs: evidence from wing form and bone strength

**DOI:** 10.1038/s41598-021-96190-1

**Published:** 2021-08-17

**Authors:** Darren Naish, Mark P. Witton, Elizabeth Martin‑Silverstone

**Affiliations:** 1 School of Biological Sciences, Faculty of Environment & Life Sciences, University of Southampton, University Road, Southampton, SO17 1BJ UK; 2 School of the Environment, Geography and Geosciences, University of Portsmouth, Burnaby Building, Burnaby Road, Portsmouth, PO1 3QL UK; 3 School of Earth Sciences, Life Sciences Building, University of Bristol, 24 Tyndall Ave, Bristol, BS8 1TH UK

Correction to: *Scientific Reports* 10.1038/s41598-021-92499-z, published online 22 July 2021

The original version of this Article contained errors.

In the Discussion section,

“The flight adaptations of hatchling pterosaurs cast doubt on the idea that pterosaur growth rates changed in response to the onset of powered flight^12^.”

now reads:

“The flight adaptations of hatchling pterosaurs cast doubt on the idea that pterosaur growth rates changed in response to the onset of powered flight^13^.”

In the Conclusions section,

“We are doubtful of the suggestion that changing pterosaur growth rates were linked with the onset of powered flight^12^.”

now reads:

“We are doubtful of the suggestion that changing pterosaur growth rates were linked with the onset of powered flight^13^.”

The original Article has been corrected.

